# Determination of the lactose content in low-lactose milk using Fourier-transform infrared spectroscopy (FTIR) and convolutional neural network

**DOI:** 10.1016/j.heliyon.2023.e12898

**Published:** 2023-01-10

**Authors:** Daniela C.S.Z. Ribeiro, Habib Asseiss Neto, Juliana S. Lima, Débora C.S. de Assis, Kelly M. Keller, Sérgio V.A. Campos, Daniel A. Oliveira, Leorges M. Fonseca

**Affiliations:** a School of Veterinary Medicine, Universidade Federal de Minas Gerais/UFMG, Belo Horizonte, MG, Brazil; b Federal Institute of Mato Grosso do Sul, Três Lagoas, Mato Grosso do Sul, Brazil; c Department of Computer Science, Universidade Federal de Minas Gerais/UFMG, Belo Horizonte, Minas Gerais, Brazil; d Ezequiel Dias Foundation (FUNED-MG), Belo Horizonte, MG, Brazil

**Keywords:** Low-lactose, Milk, Artificial neural network, Convolutional neural network, Deep learning

## Abstract

Demand for low lactose milk and milk products has been increasing worldwide due to the high number of people with lactose intolerance. These low lactose dairy foods require fast, low-cost and efficient methods for sugar quantification. However, available methods do not meet all these requirements. In this work, we propose the association of FTIR (Fourier Transform Infrared) spectroscopy with artificial intelligence to identify and quantify residual lactose and other sugars in milk. Convolutional neural networks (CNN) were built from the infrared spectra without preprocessing the data using hyperparameter adjustment and saliency map. For the quantitative prediction of the sugars in milk, a regression model was proposed, while for the qualitative assessment, a classification model was used. Raw, pasteurized and ultra-high temperature (UHT) milk was added with lactose, glucose, and galactose in six concentrations (0.1–7.0 mg mL^−1^) and, in total, 432 samples were submitted to convolutional neural network. Accuracy, precision, sensitivity, specificity, root mean square error, mean square error, mean absolute error, and coefficient of determination (R^2^) were used as evaluation parameters. The algorithms indicated a predictive capacity (accuracy) above 95% for classification, and R^2^ of 81%, 86%, and 92% for respectively, lactose, glucose, and galactose quantification. Our results showed that the association of FTIR spectra with artificial intelligence tools, such as CNN, is an efficient, quick, and low-cost methodology for quantifying lactose and other sugars in milk.

## Highlights

• FTIR and CNN are associated as a methodology to identify and quantify sugars in low-lactose milk.
• FTIR spectra used without preprocessing the data.
• Slight differences in FTIR spectra of sugars recognized by CNN.
• Artificial Neural Network proposed as a simple, fast, low-cost method to ensure food quality.

## 1 Introduction

Lactose is the major sugar naturally present in milk, and has a great nutritional importance as an energy source and improving vitamins, calcium and magnesium absorption. Low glycemic index, decreased cariogenic risk, and prebiotic are other potential benefits [1]. However, lactose is not well absorbed by most of the world population, due to low lactase enzyme production in the small intestine; the consequence is lactose intolerance [2]. The lactose hydrolysis of milk in the dairy industry is an alternative for people with such intolerance. However, lactose hydrolysis levels must comply to legal requirements [3].

The usual methodology for sugar estimation in milk after lactose hydrolysis is the high performance liquid chromatography (HPLC) [[4], [5], [6]]. The HPLC-RID (refractive index detector) is an efficient method to quantify monosaccharides, disaccharides and oligosaccharides in milk [7,8]. Until today, accepted methods for sugars in milk and milk products using chromatography do not apply to lactose levels below 1 mg g^−1^ [4,9].

Mid-infrared spectroscopy is one of the most used methods for analyzing milk and dairy products [10,11] and several aspects favor the FTIR use for the milk quality control, especially its potential as a screening method to detect adulteration [12]. Moreover, FTIR has high analytical output, minimum sample preparation, high sensitivity, and low operating cost [13–17]. Nevertheless, the absorption wavelengths for lactose, glucose, and galactose are quite similar [16,17], requiring the use of new analytical tools, in order to ensure precision and selectivity to analyze residual lactose in low-lactose products.

Since a large amount of data is obtained using FTIR for milk analysis [18], these data patterns can be used to learn and predict trends. Machine learning (ML) algorithms have been widely used for different applications due to their potential use to describe and explain hidden phenomena in the data set, and to predict new values [19–22].

Supervised learning can be divided into regression and classification models. In regression, the output is always a quantitative and continuous variable. Conversely, for classification models, the expected output is a categorical variable (dependent variable) as a function of the input (independent variables) [23].

In the context of machine learning, Convolutional Neural Networks (CNN) are Neural Network architectures that have become important tools in several areas addressing image and video pattern recognition, speech recognition, text analysis, language processing, among others [20,21]. CNN are inspired by the visual processing of the human brain and have additional layers when compared to traditional neural networks. They are applied to image data, in two-dimensional arrays, which detect edges and other features of the input; however, the same idea is applied to one-dimensional data, such as spectral data [24].

The application of neural networks in the food industry has been studied in the last years to assess product quality, integrity, geographic origin and shelf-life [25,26]. Neural networks have been used together with other tools, such as FTIR, to investigate the authenticity of milk [13–15].

In this work, the association of FTIR with machine learning tools is an innovative proposal that has not been performed to date to identify and quantify residual lactose and other sugars in low-lactose milk. Different researchers have used machine learning for classification purposes (Table 1), while in our work these tools were also applied for quantification. Our purpose was to determine the amount of the different sugars in milk. This is a potential method to obtain accurate milk composition in milk quality laboratories.

**Table 1:** Use of machine learning tools associated with FTIR, in milk samples, and their main purpose.

Method Machine Learning	Sample	Purpose	Algorithm model	References
ANN	Buffalo's milk	Adulteration screening	classification	Silva et al. [13]
CNN	Raw milk	Adulteration screening	classification	Asseis Neto et al. [14]
ANN	Raw milk	Adulteration screening	classification	Conceição et al. [15]
SIMCA	Milk powder	Adulteration screening	classification	Karunathilaka et al. [12]
CNN	Raw, pasteurized and UHT milk	Milk composition (sugars)	classification and quantification	Our research

ANN: artificial neural network.

CNN: Convolutional neural network.

SIMCA: Soft Independent Modeling of Class Analogy.

UHT: ultra-high temperature.

## 2 Materials and methods

### 2.1 Spectroscopy FTIR

**Fig. 1:**
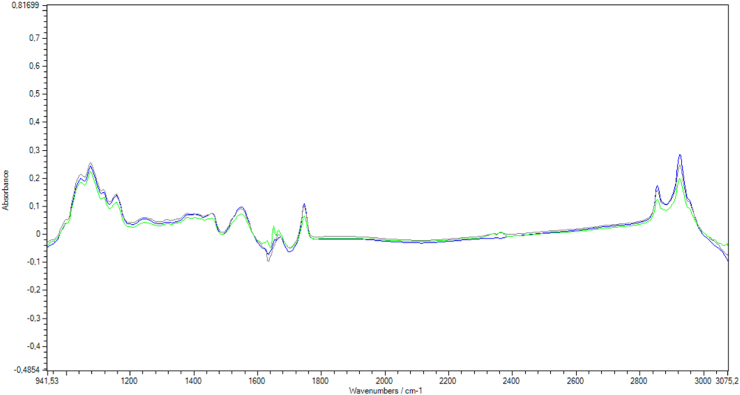
FTIR spectra of raw milk (green line), pasteurized milk (blue line), and UHT milk (grey line). (For interpretation of the references to color in this figure legend, the reader is referred to the Web version of this article.)

Raw, pasteurized and UHT milk were added of lactose, glucose, and galactose, separately (Sigma-Aldrich, USA), in six concentrations (0.1, 0.5, 1.0, 3.0, 5.0, and 7.0% (w/v)), and six batches, with a total of 108 samples added with sugar and 36 samples with no sugar addition for each milk (Fig. 1).

The raw milk was collected from refrigerated bulk tank, from March 2020 to February 2021, in an experimental research farm, with a herd of about 100 lactating cows with different genetic ratio of Holstein and Gyr cattle. According to Resolution CNS 466/2012 UFMG, this research was exempt from registration at the Research Ethics Committee (CEP/UFMG). The pasteurized and UHT milk were obtained from local retailers.

**Fig. 2:**
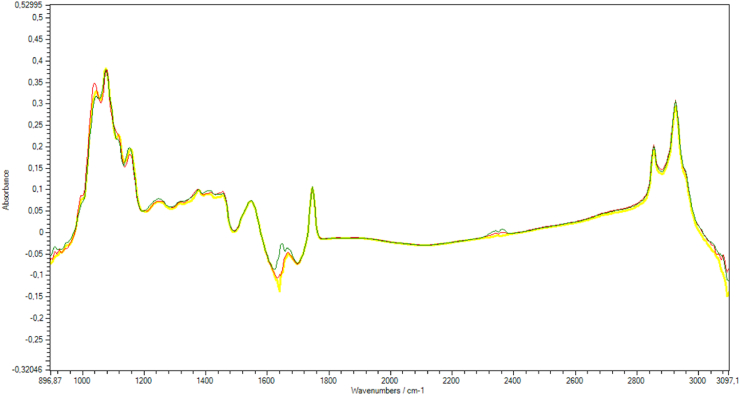
FTIR spectra of raw milk added with lactose (yellow line), glucose (red line), and galactose (green line), in concentrations of 30 mg mL^−1^. (For interpretation of the references to color in this figure legend, the reader is referred to the Web version of this article.)

Milk composition was analyzed by FTIR (LactoScope™ FTIR 400, Delta Instruments, Drachten, The Netherlands), on the Milk Quality Analysis Laboratory, School of Veterinary Medicine, UFMG (ABNT NBR ISO/IEC 17025 accredited). For each sample an infrared spectrum was generated (SPC format, Thermo Scientific, Galactic Grams). In Fig. 2, an example of infrared absorption spectra of sugars in milk, in the region of 900–3000 cm^−1^.

### 2.2 Convolutional neural network

#### 2.2.1 Analysis of infrared spectra using deep learning

**Fig. 3:**
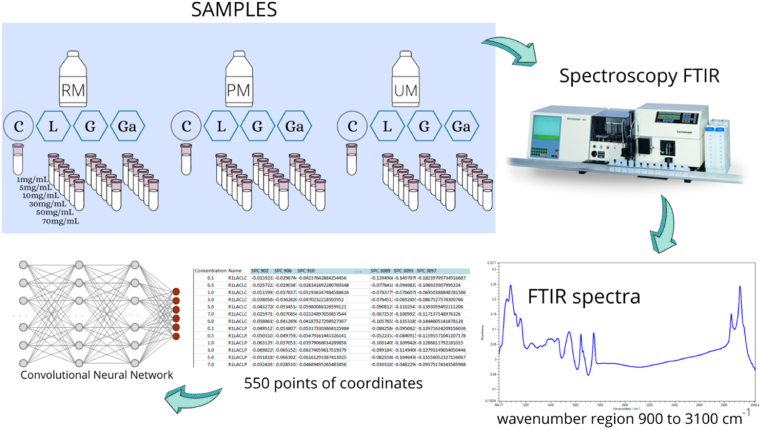
Sequence of processes from milk samples (RM = raw milk; PM = pasteurized milk; UM=UHT milk) to CNN using FTIR spectra as input features (C = control; L = lactose; G = glucose; Ga= Galactose).

FTIR spectra of the 432 milk samples were used as input features for the proposed CNN. These coordinates were composed of 550 points and represent the absorption region of the spectrum from 900 to 3100 cm^−1^, with a resolution of 4 cm^−1^ (Fig. 3). The CNN interprets the spectrum of the milk sample as an “image”, as well as the convolutional layers of the filter select important characteristics of the spectrum [24].

CNN architectures were applied using Keras [27] and TensorFlow [28] in Python. Dataset readings, cross-validation and other operations were implemented using Scikit-learn library [29], based on the methodology from Asseiss Neto et al. [14] with adjusted hyperparameters.

CNN was executed with a unidimensional convolutional layer, in order to receive spectral data as input, which learns 60 filters with kernel size 20. The layer could, therefore, detect 60 features directly from the FTIR spectrum. Filters were concatenated (grouped) and followed by a denser layer (fully connected) of 2048 neurons. Auxiliary layers were used, LeakyReLU activation added non-linearity to the model [30], MaxPooling1D reduced the data through grouping, Batch Normalization operations [31] normalized the data of the layers, and Dropout operations [32] randomly ignored a certain number of neurons to offer a greater generalization power of the model, avoiding overfitting of the training data. These CNN architectures were from Asseiss Neto et al. [14] modified.

**Fig. 4:**
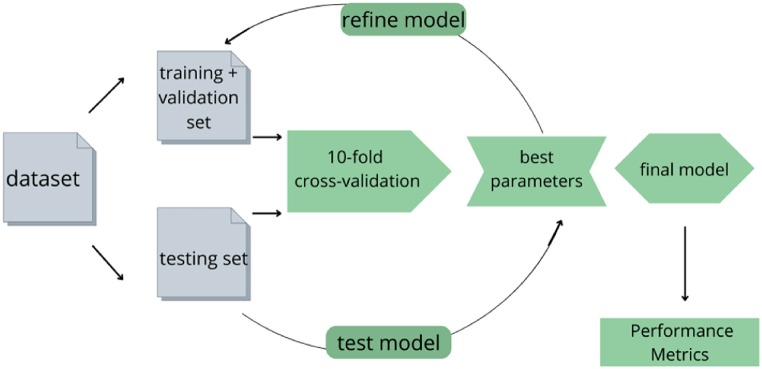
Sequential diagram for data mining.

CNN training used Adam (Adaptive Moment Estimation) Optimizer for 300 epochs for classification and 250 epochs for regression [33]. This methodology separates part of the training set into a validation set, and the performance of the model is evaluated on each epoch. In our model, 20% of the training set was separated as a validation set, during the cross-validation (Fig. 4).

#### 2.2.2 Cross-validation

**Fig. 5:**
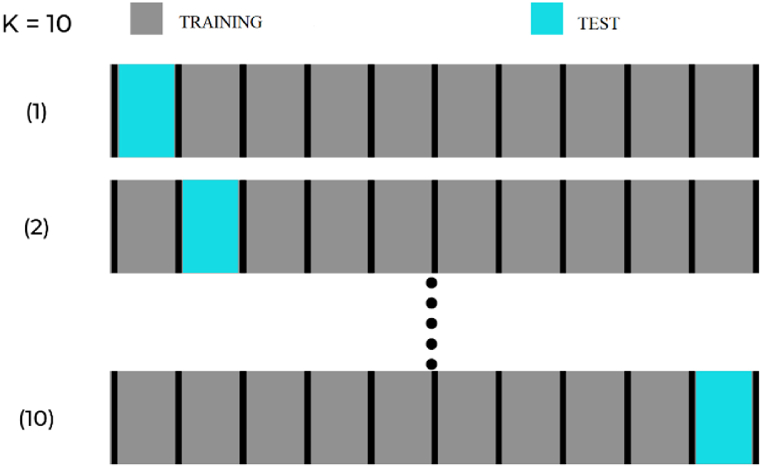
10-fold cross-validation diagram.

The *k*-fold cross-validation method was used to estimate the performance of the networks, in which the total number of cases was divided into *k* random groups, of approximate same sizes. One of the subsets was used for testing, and this process was repeated for each *k* group. At the end, an average accuracy rate was obtained from the rates of each subset [34]. The choice for the number of subsets (*k*) was 10 *k* (Fig. 5).

#### 2.2.3 CNN classifier

**Fig. 6:**
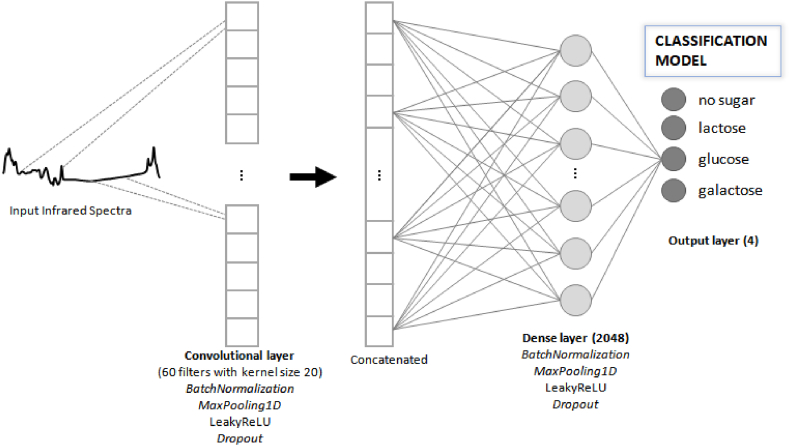
Schematic representation of a Convolutional Neural Network for classification of added sugar in milk, such as lactose, glucose, galactose, and no sugar added.

For the classification problem, a CNN was trained with the data set of milk samples with no sugar addition and with addition of lactose, glucose, and galactose, in six different concentrations, 0.1, 0.5, 1.0, 3.0, 5.0, and 7.0% (w/v). Once the samples were analyzed the model was able to recognize the spectra of each saccharide. The results of the classification models were variable responses of a categorical nature, that is, the presence or absence of a specific sugar in milk. In the output layer, there were four final neurons represented by each class to be predicted, lactose, glucose, galactose and no sugar added (Fig. 6).

#### 2.2.4 CNN for regression

**Fig. 7:**
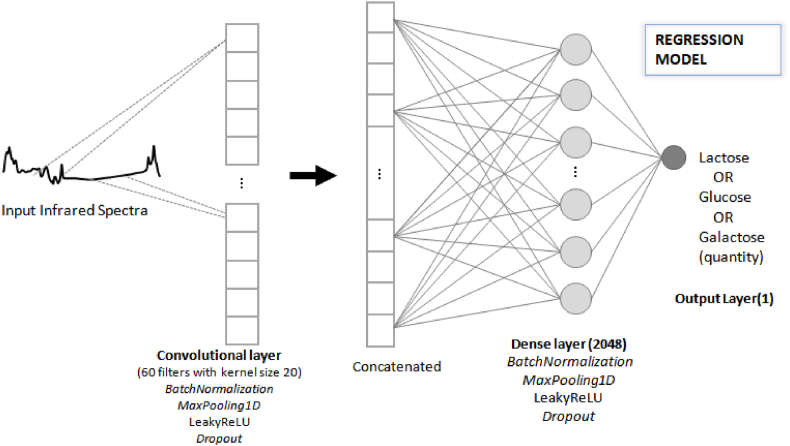
Schematic representation of a Convolutional Neural Network for quantification of added sugar in milk, such as lactose, glucose, or galactose.

The CNN trained for regression models received as input data the results of the spectra of milk samples added or not with the three sugars (lactose, glucose, and galactose), in six different concentrations, 0.1, 0.5, 1.0, 3.0, 5.0, and 7.0% (w/v). The sugars added to milk caused absorbance peaks that were correlated to the component amount in the milk [35]. The resulting regression models were continuous variable responses which corresponded to each sugar concentration in milk (Fig. 7). For quantification of each added sugar, the network changed the output layer, the final neuron. Then, three CNNs were built for regression, that is, for lactose, glucose, and galactose.

### 2.3 Statistics

Performance metrics were used to predict ability of CNN models. CNN classifiers were evaluated based on accuracy, precision, sensitivity, and specificity, where

$$Precision=\frac{true\;positive\;\left(TP\right)}{true\;positive+false\;positive\;\left(FP\right)}$$

$$Sensitivity=\frac{true\;positive}{true\;positive+false\;negative\;\left(FN\right)}$$

$$Specificity=\frac{true\;negative\;\left(TN\right)}{true\;negative+false\;positive}$$

$$Accuracy=\frac{samples\;correctly\;predicted\;\left(TP+TN\right)}{total\;samples\;\left(TP+TN+FP+FN\right)}$$

Accuracy is the proportion of cases that were correctly predicted among total cases, whether they are true positive or true negative, and it is considered the correctness rate.

The ROC (Receiver Operating Characteristics) curve and AUC (Area Under the Curve) are two metrics widely used for classification models, which assess the relationship between sensitivity and specificity [14,34]. The ROC curve was built from the true positive rate (sensitivity), on the y-axis, and the false-positive rate (1-specificity), on the x-axis.

For CNN regression, the response variable is a continuous, numerical variable, and the most common metric to characterize the model predictive abilities is the Root Mean Square Error (RMSE). RMSE (Eq. (1)) is the root regression model residual sum, which is the difference between real and predicted values, calculated with the regression equation [34].

(1) $$RMSE=\sqrt{\frac{\sum {\left({\hat{y}}_{i}-y_{i}\right)}^{2}}{n}}$$

The Mean Square Error (MSE) (Eq. (2)) is the mean of the sum of the squares of errors for each case, both for the training set and for the test set [34].

(2) $$MSE=\frac{\sum {\left({\hat{y}}_{i}-y_{i}\right)}^{2}}{n}$$

The mean of the deviations, described by the Mean Absolute Error (MAE) has the response unit equal to the unit of the input values (Eq. (3)). Its value represents the average deviation between predicted and real values. Comparing the MSE and MAE, the first gives greater weight to large deviations (as they are squared), while the MAE gives equal weight to all deviations [34].

(3) $$MAE=\frac{\sum \left|{\hat{y}}_{i}-y_{i}\right|}{n}$$

Another metric widely used in regression models is the coefficient of determination, presented as R^2^, in Eq. (4). This value expresses the proportion of information in the data that is explained by the model. The value of R^2^ ranges from 0 to 1 and is usually represented as a percentage [34].

(4) $$R^{2}=1-\frac{SSR}{SST}$$

where: In Eq. (5), SSR is the sum of squares regression; it means the variability explained by the regression. In Eq. (6), SST is the sum of squares total, and it means the total variability of the dataset.

(5) $$SSR=\sum_{i=1}^{n}{\left({\hat{y}}_{i}-\bar{y}\right)}^{2}$$

and

(6) $$SST=\sum_{i=1}^{n}{\left(y_{i}-\bar{y}\right)}^{2}$$

where: ***n*** is number of samples, ${\hat{y}}_{i}$ is predicted value, $y_{i}$ is real value, and $\bar{y}$ is yhe mean of the dependent variable.

These metrics were used to visualize the power of CNN for regression to quantify lactose, glucose, and galactose in milk, after 10-fold cross-validation.

## 3 Results and discussion

CNN was able to learn from data and produce high quality predictions without the need for any preprocessing step. The neurons of the convolutional layers were used as filters that recognize specific features within the spectral regions. Only the important spectra data are passed to the deep layers of the network. For this reason, CNN was able to receive raw spectral data, and performed the extraction of features with no manual interactions [36].

**Fig. 8:**
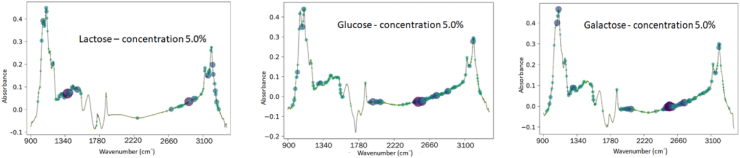
Saliency maps of the convolutional neural network with the FTIR spectra of milk samples added with lactose, glucose, and galactose (50 mg mL^−1^).

The most significant and representative input regions of the spectrum for CNN convolutional layers are identified in the saliency map [37]. The construction of saliency map in the mid-infrared spectrum of milk samples allows the extraction of the data from the most characteristic wavelengths regions that most contributed to the activation of a given output on the CNN. In Fig. 8 are the saliency maps for each sugar added to milk (lactose, glucose, and galactose) in the concentration of 50 mg mL^−1^.

### 3.1 CNN architecture

The CNN architecture that we used defined the activation layers, the convolutional layers, number of filters, kernel size, number of neurons in the dense layer and in the output layer, and resulted in a CNN as in Figs. 6 and 7. With the automated machine learning process, or AutoML, the definition of the architecture of a neural network allows the use of very accurate models, with good tuning of the hyperparameters that control the process (Table 2). Using this technique, the models become more suitable and with higher performance [38].

**Table 2:** Hyperparameters used to control the training process of CNN architecture.

Hyperparameter	Description	Value
Convolutional layer	Number of convolutional layer	1
Convolutional filter	Number of convolutional filters	60
Kernel size	Convolutional window size	20
Dense layer	Number of concatenated layer	1
Size dense layer	Number of neurons in dense layer	2048
Dropout rate	Rate of neurons ignored randomly to avoid overfitting	0.1
Max-pooling size	Reduced input resolution	4
Activation	Non-linearity function	LeakyReLU

### 3.2 CNN classifier

**Fig. 9:**
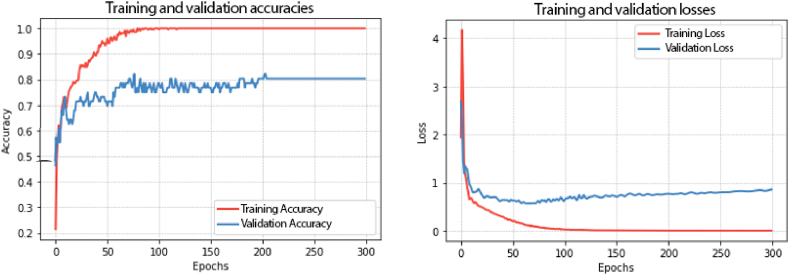
Plots of the CNN model's accuracy and loss on training and validation steps for classification of sugars added to milk, trained for 300 epochs.

During training and validation, for each epoch in the network, accuracy and loss histories were stored. These are records of each sample (Fig. 9). While epochs run, network learning evolves, accuracy increases, and losses decrease. The execution time that the training step took for the 10-fold cross-validation was 2min 7s.

**Fig. 10:**
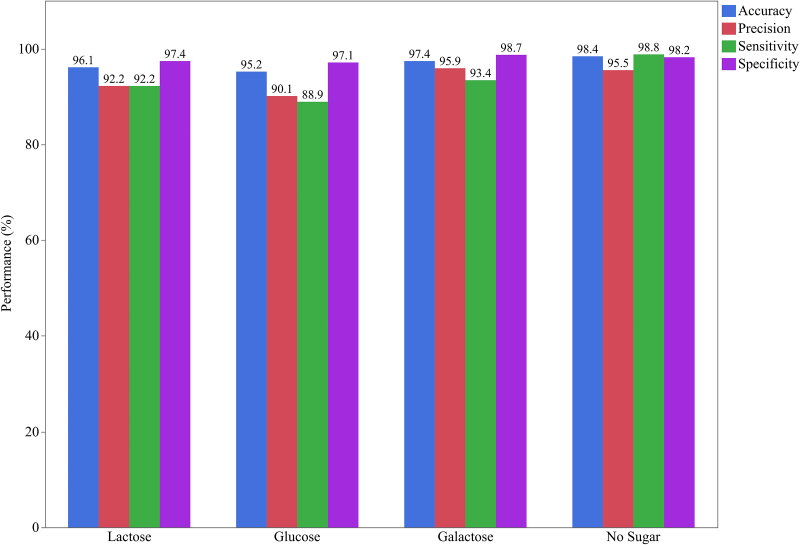
Performance metrics (accuracy, precision, sensitivity, and specificity) of CNN classifier to predict lactose, glucose, galactose, and no sugar added in milk samples.

The predictive abilities of CNN models were assessed through performance metrics. In the CNN classifier, accuracy, precision, sensitivity, and specificity after 10-fold cross-validation to predict lactose, glucose, galactose, and no sugar added were demonstrated in Fig. 10. For lactose quantification, accuracy, precision, sensitivity, and specificity were 96.1%, 92.2%, 92.2%, and 97.4%, respectively. For glucose, the metrics were 95.2%, 90.1%, 88.9%, and 97.1%, respectively. For galactose, were 97.4%, 95.9%, 93.4%, and 98.7%, respectively. For no sugar added, were 98.4%, 95.5%, 98.9%, and 98.2%, respectively. In this model, the saccharide class was predicted from four options (or four final neurons), characterizing a multiclass classification. CNN is better suited for multiclass classification on spectral data in comparison to other methods such as decision trees [14].

**Fig. 11:**
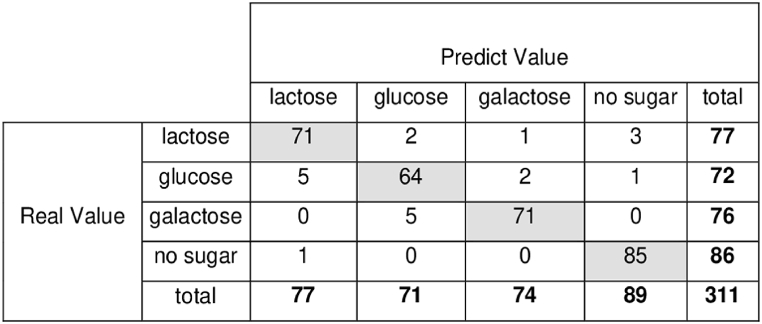
Confusion matrix to visualize the ability of CNN classifier to identify lactose, glucose, and galactose added to milk or milk no sugar added.

In Fig. 11, a confusion matrix was used to better visualize true positives (TP), false positives (FP), true negatives (TN), and false negatives (FN). These values were used to calculate the performance metrics above.

**Fig. 12:**
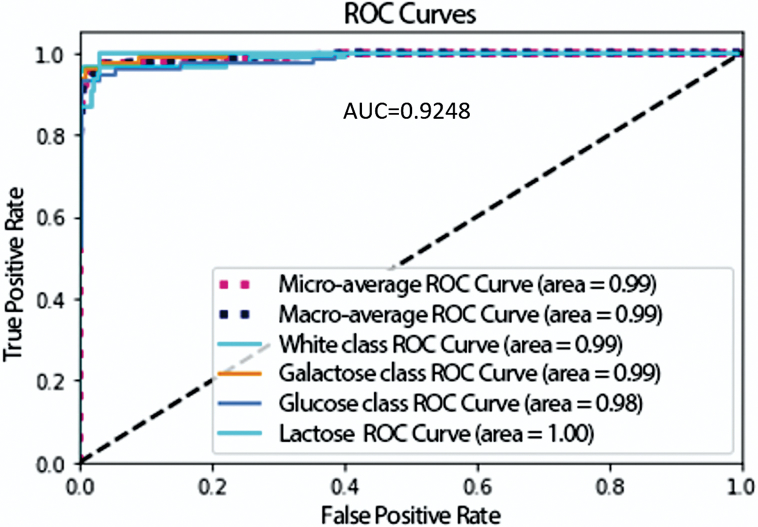
ROC (Receiver Operating Characteristics) curve and AUC (Area Under the Curve) score for multiclass classification of CNN model. The micro-average strategy aggregates values of all sugar classes and the macro-average computes values of each sugar class, lactose, glucose, and galactose. The white class is no sugar added to milk.

The receiver operating characteristics curve was an important visualization tool for the method performance. Fig. 12 shows the ROC curve of the classification model. ROC curves are defined for binary classification problems, but there are extensions of the technique that consider the problem of multiclass classification [34]. In this work, AUC score for classification was 0.925, which means that the model has high capacity to predict.

### 3.3 CNN for regression

In order to quantify lactose, glucose, and galactose, three different CNNs were used. Each neural network used the specific spectra of each sugar as input data, and the output neuron corresponded to the predicted concentration of sugar.

**Fig. 13:**
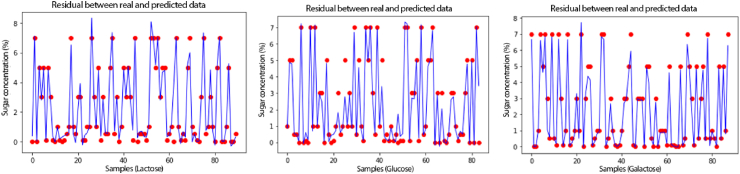
Residual plot of regression algorithms to quantify lactose, glucose, and galactose in milk. The blue line represents predicted values and red points represent real values of samples concentrations. (For interpretation of the references to color in this figure legend, the reader is referred to the Web version of this article.)

In a regression model, a single metric is not enough to understand the strengths and weaknesses of a regression model. In addition to RMSE, MSE, R^2^ and visualizations of the model's fit, particularly by the residual plot, are essential to understand whether the model is suitable for the purpose. The adequacy of the models can be observed by the residual plot between predicted and real values (Fig. 13). The predictive power of the model is related to how often predicted values approach real values.

**Fig. 14:**
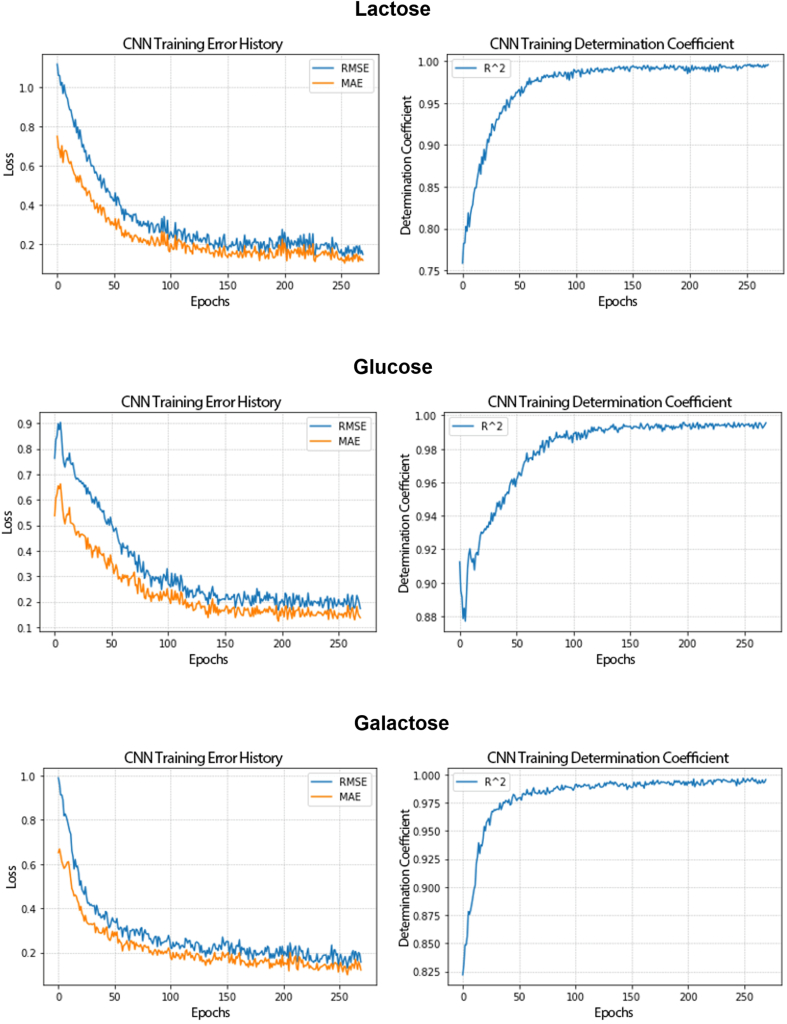
Plots of error history and coefficient of determination over CNN’s training for 250 epochs for lactose, glucose, and galactose quantification in milk. RMSE: root mean square error; MAE: mean absolute error; R^^2^: coefficient of determination.

During the training, the network has learned and decreased the error rate generated by the model. In Fig. 14 the decrease in the error history and the increase in the coefficient of determination in the training step show that the model increased its predictive capacity for 250 epochs, with the CNN for lactose, glucose, and galactose quantification. The execution time for the training step in the 10-fold cross-validation was 1min 51s.

The performance metrics of CNN for regression after 10-fold cross-validation, are shown in Table 3. If the coefficient of determination (R^2^) is close to 1, most of the variance of the predicted values can be explained by the model. In this work, the model was able to explain 91.7% of the variance of the values to galactose, 85.5% to glucose, and 81.1% to lactose.

**Table 3:** Results of CNN performance metrics to quantify lactose, glucose, and galactose in milk, after 10-fold cross-validation.

Performance Metrics	Lactose	Glucose	Galactose
RMSE	0.8646	0.7981	0.6110
MSE	0.7475	0.6369	0.3733
MAE	0.5997	0.5564	0.4748
R^2^	0.8106	0.8552	0.9177

RMSE: root mean square error, MSE: mean square error, MAE: mean absolute error, R^2^: coefficient of determination.

The construction of a CNN for regression differs from the work of Asseiss Neto et al. (2019) who used CNN to classify milk samples with five adulterants and did not use the K-fold cross-validation method. *k*-fold cross-validation method provides a more realistic view of the predictive power of models, compared to methods which split the dataset into proportions for training, testing, and validation. In cross-validation all subgroups are trained and tested, being valid in an uneven distribution of data. Previous works, using neural networks in milk, did not use cross-validation for method validation. Conceição et al. [15] used the training and validation groups in a 70/30 ratio, and Asseiss Neto et al. [14] used the training and test set in a 90/10, 75/25 or 50/50 ratio, considering 20% of the training set as the validation set.

Auto ML defined the CNN architecture with the implementation of the hyperparameters, and the results were able to quantify new values. The CNN architecture used existing libraries, but the novelty in the methodology is based on the use of these architectures for milk samples, and the model could be applied to quantify lactose and other sugars in milk.

These results indicated that the algorithm was able to recognize slight differences in the spectra of each saccharide with good precision and selectivity. Additionally, the CNN for lactose, glucose, and galactose identification and quantification in milk was a fast method, with execution time around 2 min, and low-cost.

## 4 Conclusions

Classification and regression algorithms proved to be useful to identify and quantify sugars in low-lactose milk. Especially, when CNN was applied to the spectrum of each sugar, their identification was possible, even with only slight differences in the lactose, glucose, and galactose spectra. The method proposed was efficient, selective, low-cost, and fast.

The great potential of machine learning demonstrates that better performance metrics can be reached with small network architecture changes, with a huge potential for using machine learning as a tool to quantify saccharides in milk for food quality control.

For future work, it will be interesting to consider the composition variability of milk samples, in order to guarantee the application of the proposed method under different conditions. Other point for improvement will be to apply external validation, with different samples that were not used in the cross-validation.

## Author contribution statement

Daniela C. S. Z. Ribeiro: Performed the experiments; Analyzed and interpreted the data; Wrote the paper.

Juliana S. Lima: Performed the experiments.

Habib Asseiss Neto, Sérgio V. A. Campos: Conceived and designed the experiments; Analyzed and interpreted the data.

Débora C. S. de Assis, Kelly M. Keller, Daniel A. Oliveira: Contributed reagents, materials, analysis tools or data.

Leorges M. Fonseca: Contributed reagents, materials, analysis tools or data; Analyzed and interpreted the data; Wrote the paper.

## Funding statement

Daniela Cristina Solo de Zaldivar Ribeiro was supported by Coordenação de Aperfeiçoamento de Pessoal de Nível Superior [88882.348827/2010-01].

Mr Leorges Fonseca was supported by Fundação de Amparo à Pesquisa do Estado de Minas Gerais [APQ-02740-17].

## Data availability statement

Data included in article/supp. material/referenced in article.

## Declaration of interest's statement

The authors declare no competing interests.
